# The Foundation Programme in psychiatry: a qualitative study into the effects of a foundation placement

**DOI:** 10.1192/pb.bp.115.051243

**Published:** 2016-10

**Authors:** Ann Boyle, Sophie Davies, Nisha Dogra, Jennifer Perry, Hannah Fosker

**Affiliations:** 1 Leicestershire Partnership NHS Trust; 2 CAMHS Young Persons Team, Leicester; 3 Greenwood Institute of Child Health, University of Leicester; 4 South London and Maudsley NHS Foundation Trust, London

## Abstract

**Aims and method** There is a drive to increase the number of psychiatry foundation placements to ensure that training keeps up with the changing health and social care landscape. This qualitative study aimed to explore, by interview, the experiences of 17 doctors who have completed a foundation placement in psychiatry.

**Results** The study highlights the benefits of foundation psychiatry placements and some of their positive and negative aspects.

**Clinical implications** Those developing foundation placements will need to ensure they are of high quality.

*Broadening the Foundation Programme Report*^1^ describes how England's health and social care landscape is changing. People are living for longer and with complex, chronic health conditions. They are increasingly experiencing longer periods of disability and there remains a lack of parity of esteem between mental and physical healthcare. These patterns demand changes in how healthcare is provided and the type of doctors required.

The 2012 Foundation Programme curriculum,^2^ Professor John Collins' *Foundation for Excellence* report^3^ and the Shape of Training report^4^ all anticipated these changing care needs for patients and recommended that foundation doctors develop their capabilities across a range of settings, including the community. Up until this point, foundation placements had been dominated mainly by medicine and surgery.

The target set is that all foundation doctors should undertake a community placement or an integrated placement from August 2017. Community placements include all psychiatry placements as well as placements in areas such as general practice and community paediatrics. The target set by the psychiatry taskforce is to have 22.5% of all foundation year 1 (FY1) and 22.5% of all foundation year 2 (FY2) placements in psychiatry.

## Method

### Aims

This research aims to further understand the benefits of a foundation placement in psychiatry and to lend further support to the planned increase in psychiatry foundation jobs. Much of the literature so far has looked at improving recruitment in psychiatry, however, we would argue that there are many other potential benefits to the increase in placements. This study will also look at some of the positive and negative aspects of placements and how these can be overcome.

A qualitative study design with semi-structured interviews was chosen to undertake exploratory work to further understand the perceived benefits of psychiatry placements and the positive and negative experiences of doctors who undertake them.^5^

### Sample

All FY1 and FY2 doctors who had completed a foundation placement in psychiatry in Leicestershire in two consecutive academic years were invited to participate. There were no exclusion criteria. Recruitment of participants was continued until thematic saturation was reached.

### The interview

The interview schedule was developed to explore perceived benefits and positive and negative experiences of psychiatry foundation placements. It was hoped that an individual, private interview would facilitate free, uninhibited expression by the participants.

### Procedure

A list of all eligible foundation doctors was created by the Leicestershire, Northamptonshire and Rutland (LNR) Foundation School. A researcher contacted all potential participants by email and if a positive response was received, a mutually convenient time was arranged for the interview. To look for selection bias of only recruiting those trainees who wanted to do psychiatry, we asked directly what their career intentions had previously been and what they were at the time of the interview. All interviews were taped or digitally recorded.

### Analysis

All the interviews were transcribed verbatim and thematic analysis was undertaken. Themes were identified independently by researchers before discussion to reach agreement on those of relevance to the research question. This procedure helped to ensure reliability. Themes were compared across scripts to develop conceptual ideas beyond description and these were then categorised.

## Results

We interviewed 17 out of a potential 84 doctors. In relation to benefits of psychiatry placements, the key themes identified were the opportunity to acquire knowledge of mental health which can be used across specialties, the opportunity to develop transferable skills, the potential to overcome stigma, and improved recruitment. Doctors reported that tasters, supervision, positive role models and work-life balance were key positive factors in their placements. The key negative themes were that some placements were perceived to be of poor quality and concerns about the lack of medical exposure.

### Benefits of foundation psychiatry placements

#### Opportunities to develop knowledge and experience of psychiatry which will be useful across all specialties

Participants spoke of how common psychiatric presentations were within all specialties:

‘You get patients with psychiatric problems in every bit of medicine’.

‘Often I think patients presenting with a medical or surgical problem have underlying psychopathology’.

One doctor highlighted how doctors in other specialties often ‘don't know how to deal with patients [with psychiatric problems]’. An aspiring general practitioner described how in the future:

‘I will be so much more confident at managing psychiatric problems, presenting a mental state, getting advice, assessing risk and all the other things’.

This supports the importance of psychiatric experience for all doctors, regardless of their future career intentions.

#### Opportunities to develop transferrable knowledge and skills

Participants whose career intentions lay outside of psychiatry spoke highly of the opportunity to develop ‘transferrable’ skills in a psychiatry job, with ‘communication skills’ being commonly cited as an area particularly developed. One doctor said:

‘I think [a psychiatry foundation placement] is good because it helps to develop communication skills much better. You have to sit and talk to people and you kind of get to know why things work for some people and not others […]. [I valued] being able to just go and talk to people and ask them personal questions and being able to process that and make them feel at ease at the same time’.

Placements also presented doctors with valuable opportunities to learn about ‘continuity of care’, ‘capacity’ and ‘the complexities of chronic illness’.

#### Overcoming stigma and negative attitudes towards mental health

A number of negative aspects of psychiatry were described by participants. Stigma from a wide range of sources including the media, family, peers and doctors in other specialties was explicitly identified. One doctor asked, ‘Why do you want to do psychiatry? Because you won't be able to defend yourself … if a patient is aggressive’. Another issue raised by doctors was that psychiatry did not achieve very much for its service users. Finally, participants reported having the impression that other doctors felt psychiatrists are ‘not proper doctors, they don't help anybody’, and that somehow applying to psychiatry implied ‘a failure and [that] you have just given up on all ambition’.

‘The negative image came predominately from surgical consultants … they seem to think it's a bit of a joke career’.

It seems a job in psychiatry can help to alter these misconceptions held by doctors.

One aspiring surgeon reported:

‘My attitude towards the psychiatric patients has changed a lot. I remember when I had a patient before I did the block and I really didn't know what to do. I just rang the psychiatrist and said “Look, I'm a surgeon, I don't know anything, this guy's just unwell. Can you come and have a look at him?” Whereas now I think I'd have a bit more of a mature approach, try and get an idea of what's going on and try and give a better, more informative referral’.

Another said:

‘As a med[ical] student I left my placement with a lot of misconceptions about psychiatry and doing a psychiatry job as a foundation doctor has dispelled a lot of those misconceptions’.

In reference to beliefs about psychiatry not having good patient outcomes, one participant reported: ‘You only know you can make a difference when you see it in real life’. Another said: ‘It's really good to know what people can achieve in psychiatry’.

#### Opportunities for improving recruitment

Foundation placements can also provide opportunities for recruitment into psychiatry. Before their psychiatry placement, four participants had identified psychiatry as a potential career. After the placement seven said psychiatry was the most likely career option, including the initial four (Table 1).

**Table 1:** Participants' career choices before and following a Foundation Programme psychiatry placement

Career option	Before	After
Psychiatry	4	7

General practice	6	4

Surgery	2	2

Paediatrics	2	2

Emergency medicine	1	1

General medicine	2	1

The number expressing a preference for a career in psychiatry increased slightly. The placement did not alter career intentions for those who had already decided on a career in psychiatry. Most of those committed to psychiatry commented that doing the placement had confirmed their interest, and for some the experience introduced psychiatry as a career option. One doctor said: ‘I think if I hadn't done [psychiatry] I wouldn't have thought of it as an option’, and another admitted: ‘Before I started psychiatry I would never have considered it’.

### Positive aspects of foundation placements

Participants described a number of positive aspects of their psychiatry placements.

#### Tasters

Trainees who reported that their consultants had arranged experiences (tasters) in other areas, typically the crisis resolution team and personality disorder service, reflected on these experiences positively. This supports the importance of ensuring breadth in psychiatric placements.

#### Supervision and role models

Almost all trainees were very positive about having 1 hour supervision from a consultant every week and felt valued: ‘My supervision has been excellent really … [psychiatry consultants were] more available than any of the other supervisors I have had in the past’.

Trainees whose consultants or registrars showed an interest in them reported positive experiences, which supports previous research stating the importance of good role models in psychiatry. One trainee said that in psychiatry, consultants ‘know your name, which as an FY1 is a big deal’. Another described how,

‘We had a really good consultant … he was just interested in what we thought and talked to us and let us go and talk to any of his patients. We got to go out with [community psychiatric nurses] and things like that and go to his out-patient clinics’.

Others commented that consultant psychiatrists were ‘very approachable’ and stated that they felt ‘well supported’ in their jobs.

#### Work-life balance

Lifestyle was described as a positive factor in foundation placements:

‘It seems to be fairly nice hours; you don't get quite so many patients who get acutely unwell in the middle of the night. Psychiatric patients do seem to sleep.’

Another doctor said: ‘I think you have a good work/social, lifestyle balance’.

Other positive factors noted were predictability of workload, the ability to ‘leave on time’, and the flexibility or ‘part-time work’. Not needing to come in at night as a consultant and reduced working hours were generally considered positive, although one participant noted that consultants work harder than trainees.

### Negative aspects of placements

Several trainees were critical of their placements, particularly those who felt the placement lacked variety or a specific role for the trainee. One doctor said: ‘what I feel I have done here is more of a lower-level geriatric job’.

Many of those who did not want a career in psychiatry focused on the separation from acute hospitals and missing physical medicine. The lack of practical procedures frequently came up with the phrases ‘not medical enough’ and they ‘would miss the medical side of things’.

## Discussion

It is positive to find that working within the specialty can benefit doctors regardless of their career aspirations. Doctors appreciated that mental illness is encountered in almost every branch of medicine. Psychiatry foundation training offers opportunities for trainees to develop transferable skills that are appropriate for managing the ‘whole’ patient in any setting, as well as helping to nurture doctors who are empathic, reflective and compassionate. The importance of developing such qualities in our medical workforce was highlighted in the Francis Report.^6^

The Foundation Programme curriculum^2^ delivers mental-health-specific and transferable, generic competencies. These include team-working, working across networks of care, relationships and communication with patients, and managing patients with long-term conditions. This supports the benefit of a foundation placement in psychiatry.

Psychiatry still appears to be heavily stigmatised, both from sources within and external to the medical community. This finding comes as little surprise, given the abundance of pre-existing research already reporting this.^7,8^ Our study shows that exposure to psychiatry during the early years of doctors' training is important in decreasing stigma and challenging misconceptions.

Our study found that before their psychiatry placement, four participants had identified psychiatry as a potential career and after the placement this number had risen to seven. This shows that foundation placements can help to improve recruitment into psychiatry (although it should be noted that the numbers who changed their career choice to psychiatry in this study were quite small). These findings support the literature. Kelley *et al*^9^ showed that 14.9% of those with a foundation psychiatry placement later chose psychiatry as a career, in contrast to only 1.8% of those who did not have psychiatry exposure.

Almost all of the participants interviewed were predominantly positive about their experience within psychiatry. The views expressed about supervising consultants were very positive in all but one interview, in particular stressing that the consultants were available and approachable. The importance of good role models in psychiatry has been reported in other studies.^10^ The importance of regular supervision was also highlighted by participants and this has been recognised in the Collins report, which recommends including the responsibilities of supervising foundation trainees in consultant job planning.^3^

Participants reported positive experiences of psychiatry tasters. Tasters enable further insight into the specialty and have been highlighted as a quality marker of excellent placements by the Royal College of Psychiatrists.^11^ Trainees also noted that psychiatry offered a good work-life balance, and this has been commented on in the literature as a positive aspect of psychiatry.^12^

### Study limitations

Ethics approval for the study was granted by the National Research Ethics Service (NRES). However, regional employing trusts expected us to complete their separate ethics approval process. As this was an unfunded study, this was not feasible with the resources available. We were therefore only able to sample doctors who had completed placements in one mental health trust, therefore the results may not be generalisable to other trusts in the country.

Since the research relied on participants responding to an invitation to participate by consenting to the interview, self-selection bias is likely to have occurred. It may be that those who consented were more interested in psychiatry, or equally that those who participated had particularly strong negative feelings about the placement that they were keen to express.

### Clinical implications

There is a real need to ensure that all psychiatry foundation placements are of a high quality so that doctors have an excellent experience of the specialty. Some trainees commented on poor-quality training placements and raised concerns about a lack of medical exposure. Doctors may have pre-conceived negative views about foundation psychiatry placements, which include fears about a lack of exposure to acute care or procedural experience relevant to the curriculum. Foundation doctors should be able to find opportunities to undertake procedures such as venepuncture and electrocardiograms (ECGs) during their psychiatry placements, particularly if they are based on an in-patient ward.

In conclusion, this research suggests that experience within psychiatry in the early postgraduate years can support the development of well-rounded doctors. Foundation placements in psychiatry can also help to improve recruitment to the specialty, although it should be noted that the numbers from this study were small. Improving recruitment is not the main aim of the programme but it is instead a desirable by-product. The potential to improve generic transferrable capabilities, reduce stigma and influence attitudes towards mental illness in newly qualified doctors is of vital importance. It is critical that the foundation placements are of high quality to ensure that their potential is maximised.
